# Breathing signatures of semantic and phonemic verbal fluency and their impact on test performance in a sample of young Norwegian adults

**DOI:** 10.1371/journal.pone.0314908

**Published:** 2024-12-05

**Authors:** Malin Gullsvåg, Yoshihiro Itaguchi, Claudia Rodríguez-Aranda

**Affiliations:** 1 Department of Psychology, Faculty of Health Sciences, University of Tromsø, The Arctic University of Norway, Tromsø, Norway; 2 Department of Psychology, Keio University, Minatoku, Japan; University of Aizu, JAPAN

## Abstract

Verbal fluency (VF) represents an important aspect of intelligence, in which oral word generation is demanded following semantic or phonemic cues. Two reliable phenomena of VF execution have been reported: A decay in performance across 1-minute trial and a discrepancy score between the semantic and phonemic VF tests (VFTs). Although, these characteristics have been explained from various cognitive standpoints, the fundamental role of speech breathing has not yet been considered. Therefore, the present study aims to evaluate the role of respiratory function for word generation in VFTs in healthy individuals. Thirty healthy young adults performed VFTs during definite periods of 1 minute while wearing a pneumotachograph mask. Duration, peak and volume of airflow were acquired during inspirations and expirations. Also, respiratory rate and acoustic data of verbal responses were registered, and accuracy scores were calculated. Each 1-minute trial was divided into four intervals of 15-seconds where parameters were calculated. Repeated measures ANOVAs and repeated measures correlations were used in the statistical analyses. Data revealed that respiratory function was significantly coupled to VF performance mostly during inhalations. Small but constant increments of inhale airflow occurred in phonemic VFT as well as higher peak airflow in both tasks, being higher for semantic VFT. High respiratory rate characterized performance of both VFTs across intervals. Airflow adjustments corresponded to better VF accuracy, while increments in respiratory rate did not. The present study shows a complex interplay of breathing needs during VF performance that varies along the performance period and that notably connects to inspirations.

## Introduction

Verbal fluency (VF) is the ability to produce rapidly as many words as possible according to certain rules such as belonging to a category or starting with an initial-letter. This mental capacity has been studied within broad areas of psychological research including, intelligence [1], cognitive assessment [2], brain imaging [3], aging [4] and clinical neuropsychology [5]. Besides research devoted to the clinical application of VF tasks, a major endeavor has been to understand the factors modulating normal VF performance and its neural mechanisms. It is evident that much of the knowledge about the brain mechanisms underlying VF execution stems from studies related to the BOLD hemodynamic response [6] or to oxygenation changes as reflected in the fNIRS signals [7]. These imaging techniques rely on the use of oxygen required for neural activity, which is supplied through the blood vessels and inhaled during respiration.

However, the act of breathing, that is taking air out and in of the lungs, is regarded in imaging studies as an undesirable noise disturbing brain signals evoked by neural activity [8]. Such noise also coming from other physiological functions (e.g., heart rate) has been as a rule mitigated or controlled for to understand “real” neural mechanisms (e.g., [9]). The assumption that physiological functions represent undesirable noise affecting neural signals during cognitive deployment has been contested [10]. The argumentation is that such standpoint not only hinders our understanding of the cerebral mechanisms taking place during assessment of cognitive activity, but most importantly, it biases how we regard the body and mind interaction [11].

It might be true that limitations in imaging methods have driven the idea that artifacts coming from physiological functions are noise of no relevance [12]. Still, the standpoints of what noise is or not, are not only rooted in the methods, but in the conceptualization of what brain function is [10]. As remarked by Huk & Hart (2019), to understand which factors are actually noise, researchers need to prove how the organism interacts with the purportedly noise sources and how these sources separate from controlled behaviors [11].

Based on the above, we wish to cast light on a physiological process that is regarded in various research lines as a source of noise for neural activity: the role of respiration on VF performance. Besides the need to clarify whether respiration is noise or not for this verbal ability, there are further points of interest motivating an assessment of the interplay between respiration and VF.

## Why should we examine respiration during VF performance?

Verbal fluency is customarily tested orally, which requires breathing resources for speaking, that is, for the act of vocalization. However, VF ability has proven to require significant breathing resources beyond those related to voicing. In a recent publication from our laboratory where we assessed the association between airflow needs in different verbal tasks with varying difficulty level [13], we demonstrated that semantic and phonemic VF tasks (VFTs) entailed upregulated respiratory needs. Among the evaluated verbal tasks, VFTs performance was coupled to the highest increment in volume and depth of breathing. The assessment was conducted by measuring airflow parameters during the first 15 seconds of the selected tasks. We concluded that VFTs, being the task with the higher cognitive requirements, engaged unique respiratory adaptations consisting in upregulation of the volume and peak of airflow needed. This finding opens further questions about the role of respiration in two key aspects of VF performance, namely a) on the decay over time in word production and b) on the discrepancy in output size between semantic and phonemic VF variants related to difficulty level.

### Decay in word production during VF performance

Our recent findings bring up the question of whether respiratory requirements remain or not unchanged all along the standard VF performance of 60 seconds. Because earlier research has proved that a decay in word production as a function of time occurs in both semantic and phonemic VFTs, it seems reasonable to raise the issue of how respiratory needs relate to the decline in word production in these tasks.

Several studies have documented a reduction in the number and frequency of words generated in VFTs after the first 15 seconds of performance. Word generation beyond this duration, considerably declines and flattens out in the last 30 seconds of the test [14]. This phenomenon of the decay in word generation has long been established as a hallmark of VF execution [15]. The reasons for the decay remain speculative and most of the explanations have been related to other cognitive limitations, such as exhaustion in the access of high-frequency words [15], differences in automatic vs control processes for lexicon retrieval [16] and restrictions in executive functions [17].

Thus, the reasons as to why word decay occurs are related to linguistic and psychological variables. Notwithstanding, additional factors need to be explored such as the physiological machinery supporting speech generation. Since respiration is the system most intrinsically related to oral language, its role in word decay should be addressed. Taking into account our recent findings, we suggest that respiration also varies beyond the first 15 seconds of VF performance as a function of time. However, the way in which this variation may take place is not evident. We reasoned three possible scenarios: a) respiratory needs may increase proportionally as a result of cognitive effort during the 1-minute test execution, b) airflow requirements decrease in parallel to word production and c) no changes exist in airflow requirements. These alternatives lead to an interesting issue in the cognition-breathing association, namely, the directionality of the respiratory-cognition relationship on one specific cognitive task. Until recently, it was unclear whether execution of any specific cognitive task engaged definite respiratory requirements [18] and thus, the issue of directionality was far from possible to address. Nevertheless, we have demonstrated that such specific requirements exist for verbal fluency, and hence, we are now able to address the issue of directionality.

If the first scenario is correct, it would indicate that higher airflow requirements already observed during the first 15 seconds keep raising together with cognitive effort. Since the starting period of word generation can be considered the easiest one due to the high availability of words, the rest of the time is more demanding in terms of mental and physiological effort. Under such circumstances, the cognitive demands might be exerting an effect on respiration. In the second scenario, a possible decrement in respiratory requirements might suggest a limited involvement of respiration in the cognitive effort beyond what is required for voicing. Finally, the third alternative showing no differences in airflow requirements along the 1-minute trial would suggest a unique turn-on in the disposition of air requirements that remains stable from the beginning of task execution to the end. Such a situation would imply a physiological adaptation to initial environmental demands that are unrelated to ongoing cognitive constraints.

### Discrepancy in output size between phonemic and semantic VFTs

The second aspect of VF ability that can be related to respiratory function refers to a well-known discrepancy in output size between VF variants. It is the case that during semantic VFT the number of generated words is always greater than during phonemic VFT in healthy populations [19]. This difference is explained by how the human lexicon is organized [20]. In daily life, healthy individuals produce words based on semantic associations, and therefore, performance during semantic VFT more closely resembles a natural way of word retrieval that entails rich production of words. For this reason, it is proposed that semantic fluency is easier to perform than phonemic fluency. When a subject is required to generate words according to initial letters, an unusual search of words takes place, which poses higher constraints to memory retrieval. Moreover, during phonemic VF, further restrictions are imposed, such as interdictions to say variants of a word, proper nouns and repetitions. All the latter increases task difficulty and limits the number of retrieved words in phonemic fluency. In fact, it is suggested that the average number of words that healthy young/middle-aged adults produce during phonemic VF is about 12 per minute, while the corresponding output in semantic fluency is of 20 words per minute [19, 21].

Since phonemic VFT is more difficult than semantic VFT, there is a possibility that the discrepancy output between VFTs is equally reflected on respiratory patterns. Several lines of investigation give us support for this hypothesis. To begin with, it has been recurrently reported that higher energy expenditure exists when higher mental effort is demanded [18]. Also, transient oxygen administration has shown to enhance mental functions including verbal abilities [22]. These findings suggest that increments in respiratory rate and depth during high cognitive effort may exert a role on arterial oxygenation that modulates neural activity [23]. A similar conclusion rises from studies on arousal demonstrating that higher metabolic demands occur, in terms of generalized activation of the body, as a function of task difficulty [24, 25]. Based on all the above, we expect that airflow requirements would be higher during execution of phonemic VFT.

## Method

### Participants

Thirty healthy young adults (16 women, 14 men between 21 and 33 years of age) were recruited and tested at the University of Tromsø. Recruitment took place from June 1^st^ 2021 to February 4^th^ 2022. All participants were native Norwegian speakers living in Northern Norway. Recruitment strategies included distribution of flyers with information of the study at the University campus, as well as information via social media channels and word of mouth. Inclusion criteria comprised being right-handed, Norwegian as native language, and good health status (self-reported). Participants were screened for signs of depression with Beck’s Depression Inventory revision II (BDI-II; [26]) and their lexical level was acquired with the Vocabulary sub-test of the WAIS-IV [27]. In total, five participants were excluded due to scores >13 on BDI-II (n = 2), missing data (n = 1) or because of respiratory ailments (n = 2). The present data are part of a larger project about respiratory function during speech generation in various verbal tasks. Results about the main project have been published elsewhere with the same sample of participants [13]. The present study will scrutinize results of the two verbal fluency variants across the complete testing time of 1 minute, which was not addressed in the mentioned paper. All participants were aware that participation in the study was voluntary and each of them provided written informed consent prior to the testing and interview session. The study was approved by the local Regional Committee for Medical and Health Research Ethics (REK).

### Verbal fluency tasks

*Semantic* verbal fluency was assessed following the standard procedure of asking participants to generate as many exemplars as possible of the categories “animals” and “fruits & vegetables” for one minute. The selected categories have been recurrently used in studies (including our own investigations) assessing semantic VF by means of large straightforward categories (e.g., [28, 29]. Participants were instructed to say aloud, as many possible words matching these categories, as fast as they could, without producing repetitions.

*Phonemic* verbal fluency was tested with an adaptation of the “Controlled Oral Word Association Test” (COWAT) [30] and the letters “F” and “S” were selected for the study. Although, the traditional set of letters used in the COWAT are “F”, “A”, “S”, we restricted the analysis to the two mentioned stimuli since internal reliability across these letters is high [5]. The latter also applies to the Norwegian language as strong similarities exist in letter frequencies between Norwegian and English (94% correspondence) [31]. In addition, according to a recent study [32], the proportion of words beginning with “F”, “A”, “S” in Norwegian is about 8.8%, 4.8% and 13.4% respectively. Based on the above, the selected letters “F” and “S” better allow for easiness in word generation. As with the semantic VFT, participants were asked to produce as many words as possible, as fast as they could beginning with the selected letters for one minute. Furthermore, participants were instructed to avoid repetitions, proper names, and variants of a word (e.g., book, bookshelf).

### Protocol for presentation of VFT and acquisition of answers

For both VFTs the target stimuli, either categories or letters, were presented on a 19-inch computer monitor at a distance of about 50 cm from the subject. To this purpose, we used the E-prime computer program (Psychology Software Tools, Pittsburgh, PA, USA). Before stimuli presentation, instructions were given both orally and written to all participants as well as examples with different letters and categories to confirm understanding of the rules. Stimuli for both VFTs were presented on the computer screen for the entire minute of each trial. Audible answers were recorded simultaneously during acquisition of respiratory parameters for later inspection.

### Assessment and data acquisition of respiratory function

#### Basics about speech breathing

Speech breathing is characterized by quick, short inspirations followed by larger exhalations where vocalization occurs [33] and different characteristics can be measured. In the present study, we focus on the assessment of aerodynamic properties of voicing, i.e., airflow parameters, which are commonly registered for the analysis of continuous speech [34]. Although, there exist normative data for airflow measurements during speech (e.g., [35]), their relationship to a spoken word or utterance is context-dependent and quite variable [36]. Thus, due to the huge variability in speech breathing outcomes that are task-dependent [37], the exact way in which aerodynamic measurements unfold in VFTs is difficult to assert. However, based on our precedent work, where volume and peak distinguished VF from other verbal tasks, we expect that these variables will also show adjustments along the 1-minute test trial and possibly will also be more relevant for one of the VF tasks.

### Apparatus and measurements

**Fig 1:**
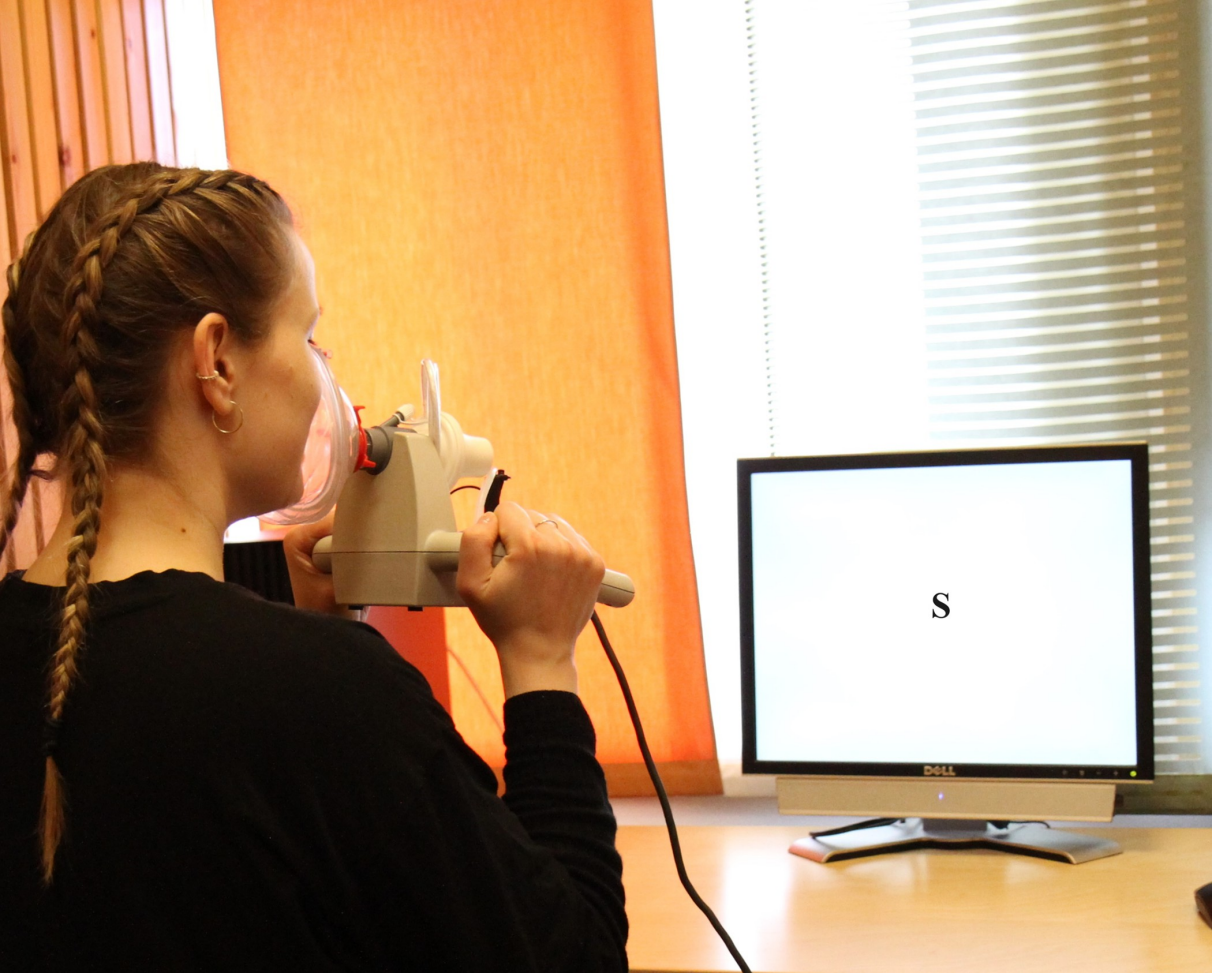
Experimental setting. A participant performing the experimental task with the pneumotachograph mask.

To acquire simultaneously acoustic and aerodynamic data during the inspiration and expiration phases of the breathing cycle, a pneumotachograph (Kay Elemetrics Phonatory Aerodynamic System (PAS), model 6600, KayPENTAX Elemetrics, Lincoln Park, NJ) was employed. This device consists of a face mask with an integrated microphone placed at 15 cm distance from the mouth (see Fig 1). The aerodynamic parameters acquired were airflow duration, peak airflow, and airflow volume. In addition, the rate of respiration was also calculated. As for the acoustic data, all recorded information was inspected at a later time to evaluate accuracy, errors, and repetitions on the answers from each participant. Though, for the present study, only accuracy data is used. An example of the acoustic and airflow data during execution of VFT can be found in the Appendix (S1 Fig).

### Protocol for acquisition of respiratory function

Participants were seated in front of the computer screen and asked to place the pneumotachograph mask on their faces by covering mouth and nose comfortably. Prior to the testing of VFTs, participants performed a vital capacity test assessing lung function (results reported in [13]), which was useful to familiarize them with the equipment. For data acquisition in the present study, all participants were instructed to hold the device tightly attached to their faces in order to avoid the risk of air leakage, while generating answers aloud on each VFT. Execution of these instructions was followed-up by the experimenter who confirmed no air leakage visible in the airflow recordings. The device was regularly calibrated before testing each participant and within each session.

### Calculation of accuracy and respiratory parameters

During the 1-minute execution of each category and letter in both VFTs, acoustic and respiratory data were acquired. Calculation of acoustic and respiratory measurements was carried out by partitioning the 1-minute trial into four 15-seconds intervals. On each interval the different parameters were calculated. For the acoustic data, the total number of correct words per letter and category was accounted. For the airflow data, duration, peak, and volume of airflow at both phases of the respiratory cycle were calculated. Rate of respiration was estimated by counting the number of speech breathing cycles in each interval. In this regard, each speech breathing cycle was counted on the visualized waveform by identification of the onset and offset locations of the breathing phases. Since two letters and two categories were used for each VFT, mean values were calculated on each interval for the semantic (mean correct words for animals and fruits & vegetables) and the phonemic tests (mean correct words for “F” and “S”). The same strategy was applied to the airflow data and respiratory rate in which the averaged results by interval were calculated.

### Rationale for the assessment of airflow variables

In contrast to studies analyzing respiration in connected speech through the “breath group” approach, we deemed necessary to assess airflow parameters based only on the breathing phases (inhalation/exhalation) during each 15-second interval. The “breath group” approach is used to assess the relationship between breathing and spoken outcomes by looking into groups of words per expiration during meaningful connected speech [38]. Since speech generation during VF is spontaneous and unpredictable, like in conversation, we considered it as continuous. However, VF is not *per se* connected speech, since subjects do not produce words relying on syntactic or grammatical rules, which are essential parts for conducting the “breath group” method [33]. Data partitioning based on 15-second intervals adheres to conventional time-course analysis of VF tasks [15].

### Procedure

Participants were tested at the department of Psychology at the University of Tromsø. Written and oral information about the study and procedures were provided to each participant prior to the testing session. Background information and other demographics were collected at the beginning of the testing session. Since evaluation of VFTs pertained to a larger project, the total time of testing was of 1.5 hours including assessment of additional verbal tasks and a cognitive battery that are not related to the present study. Due to the prerogatives of the main project, a fix order in the presentation of verbal tasks was determined in which phonemic VF was always presented firstly, followed by semantic VF.

### Statistical analyses

Descriptive statistics expressed by mean and ±SD were calculated for all variables to illustrate the sample characteristics. A series of two-factors repeated measures analyses of variance (ANOVA) were carried out with two levels of condition (semantic VF, phonemic VF) and four levels of intervals (0–15 sec., 16–30 sec., 31–45 sec., 46–60 sec.). Data were checked for sphericity with Mauchly’s test and in case of nonsphericity, the Huynh-Feldt procedure was applied. The factorial repeated measures ANOVAs were conducted to each of the respiratory parameters (duration, peak, and volume airflow, as well as respiratory rate) and for accuracy data (correctly produced words) from both VFTs. Significant main effects of condition and intervals were examined using post hoc Tukey’s HSD tests. In the case of significant interactions, tests for simple main effects were employed. Thereafter, the within-individual temporal associations of respiratory variables and correct responses by VF task were determined at each interval by repeated measures correlation with the rmcorr package [39]. Descriptive statistics and the factorial ANOVAs were performed with IMB SPSS Statistics version 28 (IBM Corp., Armonk, N.Y., USA), while the repeated measures correlations were performed in R, version 4.22 (R Foundation for Statistical Computing, Vienna, Austria). All reported p-values, when necessary were adjusted with the Bonferroni correction.

## Results

### Demographics and sample characteristics

The sample of participants in the present study is the same one as the one reported in [13]. The sample had a mean age of 25.37 years (SD = 3.21) and 16.77 years of formal education (SD = 2.16). BDI mean score was of 3.67 (SD = 2.88), which is a value below the cut-off score indicating the possibility of depression. Finally, the participant’s lexical level assessed by the Vocabulary sub-test of the WAIS-IV, showed a mean score of 35.90 (SD = 7.35), which is in accordance with reported age mean scores for our sample [40].

### Accuracy scores by VFT

**Fig 2:**
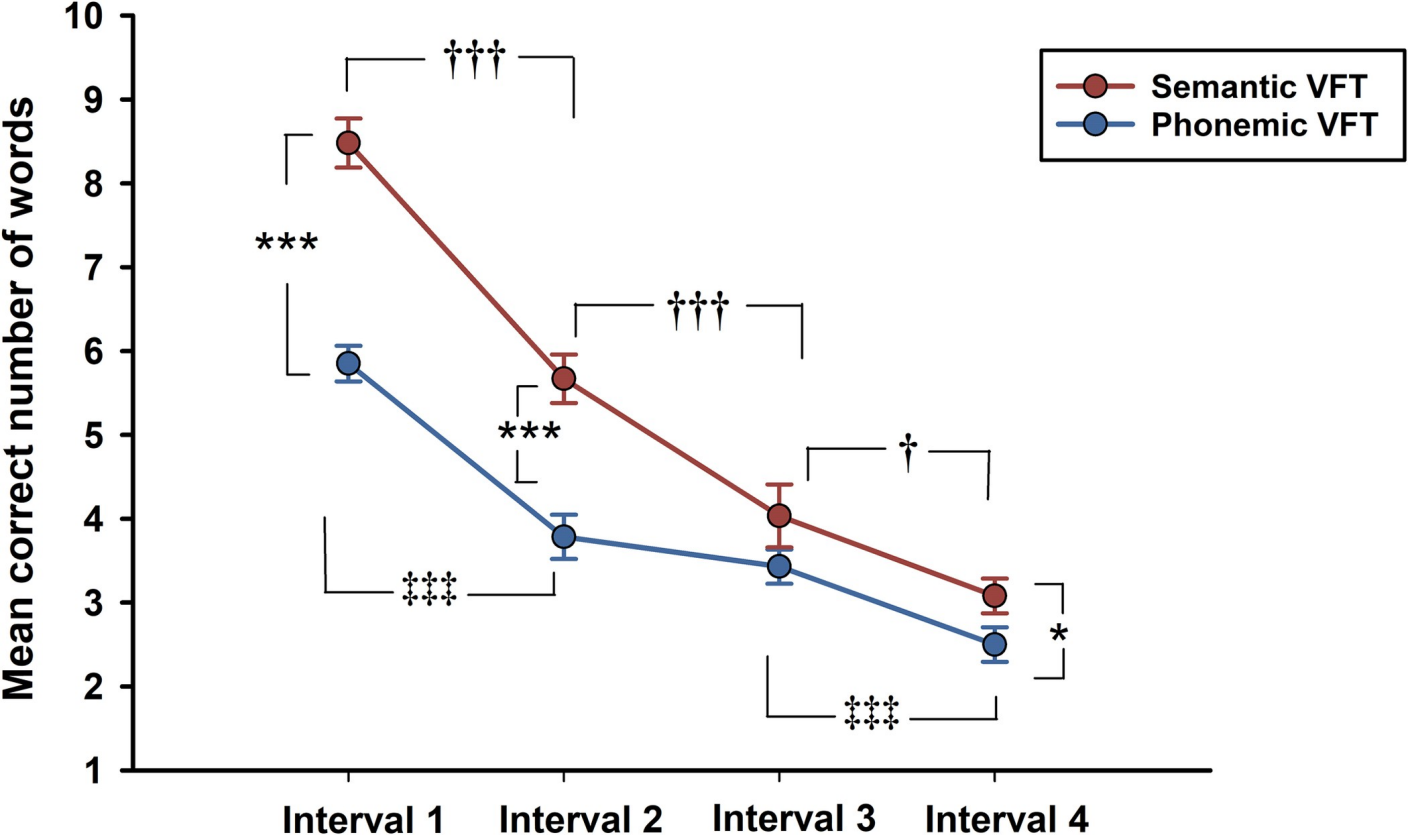
Accuracy scores by 15-seconds intervals. Mean *± SE* correct number of words per VFT by time interval. VFT = verbal fluency test. *p <* .05*; *p <* 0.01**; *p <* 0.001***. *p <* .05†; *p <* 0.001†††. *p <* 0.001‡‡‡. Significant differences between interval 1 and intervals 3 and 4 in both VFTs existed at *p <* 0.001, but they are not displayed in the graph to avoid overplotting.

The two-way ANOVA conducted for number of correctly produced words in both VFTs showed significant main effects of interval (*F* (3, 87) = 126.11, *p <* 0.001, *η*^*2*^_*p*_ = .813) and test (*F* (1, 29) = 76.02, *p <* 0.001, *η*^*2*^_*p*_ = .724) as well as a significant interaction (*F* (3, 87) = 12.55, *p <* 0.001, *η*^*2*^_*p*_ = .302). These significant results were followed by pairwise comparisons of simple main effects, which showed that word production in semantic VFT was significantly higher than in phonemic VFT on intervals 1 (*p <* 0.001), 2 (*p <* 0.001), and 4 (*p <* 0.05) (see Fig 2). No differences between tests regarding output size were found on interval 3. As for the decay in word production, the results demonstrated significant reductions in number of generated words from interval 1 to 2 in both VFTs (*p <* 0.001), from interval 2 to 3 only in semantic VFT (*p <* 0.001) and from interval 3 to 4 in both VFTs, but at different significant levels (semantic = *p <* 0.05; phonemic *p <* 0.001).

### Respiratory airflow parameters

#### Airflow duration

**Fig 3:**
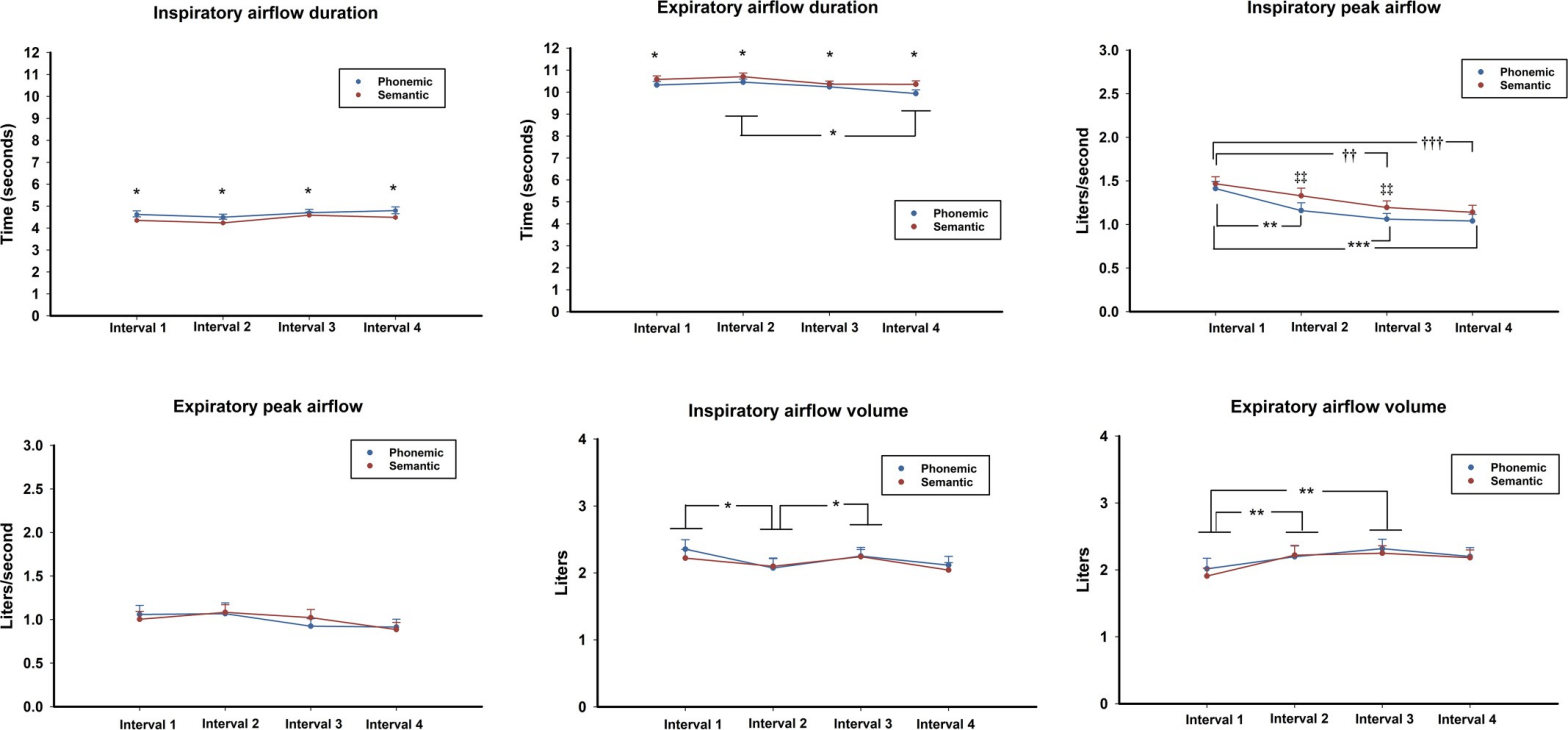
Airflow outcomes by respiratory phase. A) Mean *± SE* inspiratory airflow duration by time interval. Significant differences *p <* .05* between VF tasks across all intervals. B) Mean *± SE* expiratory airflow duration by time interval. Significant differences *p <* .05*, between VF tasks across all intervals. C) Mean *± SE* inspiratory peak airflow by time interval. *p <* 0.01**; *p <* 0.001***, refers to interval differences for the phonemic VF tasks. *p <* .01††; *p <* 0.001†††, refers to interval differences for the semantic VFT; *p <* 0.01‡‡, refers to differences between VFTs. D) Mean *± SE* inspiratory peak airflow by time interval. E) Mean *± SE* inspiratory airflow volume by time interval. *p <* .05*. F) Mean *± SE* expiratory airflow volume by time interval. *p <* .01**.

The two-way repeated measures ANOVAs applied to airflow duration demonstrated only a significant main effect for tests in the inspiratory phase (*F* (1, 29) = 4.94, *p <* 0.05, *η*^*2*^_*p*_ = .146) in which consistently, higher values existed during the phonemic task as compared to the semantic VFT (see Fig 3A). However, results during expiration showed a main effect of intervals (*F* (3, 87) = 4.43, *p <* 0.05, *η*^*2*^_*p*_ = .133) and of tests (*F* (1, 29) = 6.00, *p <* 0.05, *η*^*2*^_*p*_ = .172), but no significant interactions (*F* (3, 87) = .84, *p* = NS). The main effect for the test shows that duration was constantly longer for semantic VFT, while the main effect for intervals demonstrated differences in both tasks between the second and the fourth interval.

#### Airflow peak

For the peak airflow measurements, the two-way repeated measures ANOVAs showed significant main effects during inspiration of both intervals (*F* (3, 87) = 18.28, *p <* 0.001, *η*^*2*^_*p*_ = .387) and tests (*F* (1, 29) = 9.44, *p <* 0.01, *η*^*2*^_*p*_ = .246), with no significant interactions. These results showed a steady decline in peak airflow from interval 1 until the last interval. While this trend was present in both VFTs, the most pronounced differences were observed for the phonemic VFT. In this test, post hoc analyses demonstrated a large difference between interval 1 and intervals 3 and 4 (*p <* .001), while a significant difference also existed between interval 1 and 2 at a lesser extent (*p <* .01). As for semantic VFT, the differences appeared only between interval 1 and 3 (*p <* .01) as well as for interval 1 and 4 (*p <* .001). Moreover, results demonstrated that there were significant task differences in which the peak airflow was always higher for semantic than for phonemic VFT, most notably at intervals 2 and 3 (*p <* .01) (see Fig 3C). Interestingly, no significant results were observed for peak airflow during the expiration phase (see Fig 3D).

#### Airflow volume

Results for airflow volume showed only a main effect of intervals both during inspiration (*F* (3, 87) = 3.868, *p <* 0.05, *η*^*2*^_*p*_ = .118) and expiration (*F* (3, 87) = 6.664, *p <* 0.001, *η*^*2*^_*p*_ = .187). No significant main effect of task or interactions were found. Post hoc Tukey HSD tests showed that during the inspiratory phase, small but significant variations in volume of airflow existed, specifically on the second interval, which contrasted at *p <* 0.05 with intervals 1 and 3 (see Fig 3E). As for the volume measures during expiration (see Fig 3F), Post hoc Tukey HSD tests showed a significant increment from interval 1 to 2 and 3 (*p <* 0.01).

#### Respiratory rate

**Fig 4:**
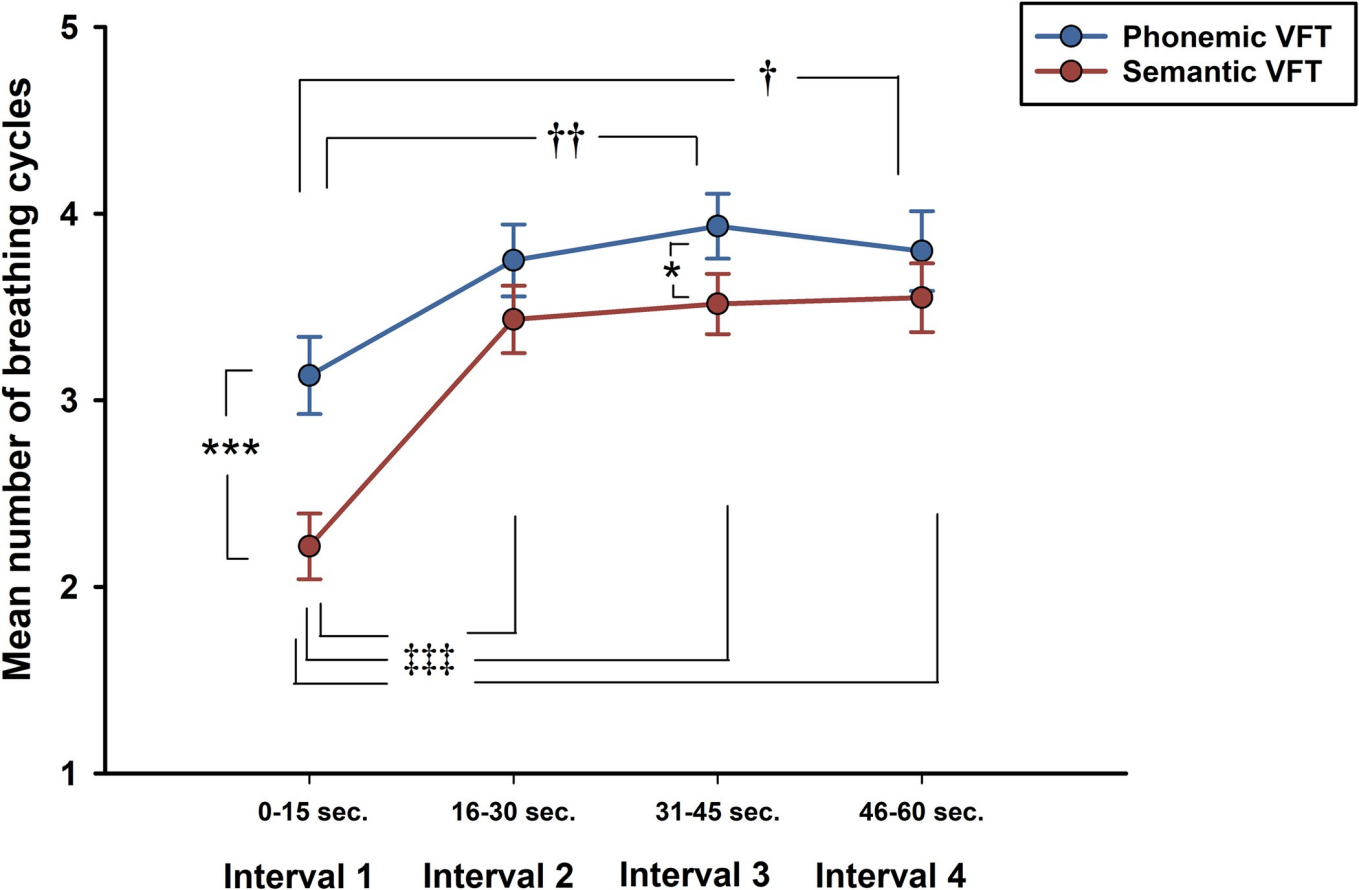
Respiratory rate by 15-seconds intervals. Mean *± SE* respiratory rate by time interval. *p <* .05*; *p <* 0.001***. *p <* .05†; *p <* 0.01††. *p <* 0.001‡‡‡.

This time the two-way repeated measures ANOVAs showed a main effect of intervals (*F* (3, 87) = 21.59, *p <* 0.001, *η*^*2*^_*p*_ = .427) and tests (*F* (1, 29) = 14.76, *p <* 0.001, *η*^*2*^_*p*_ = .337) and a significant interaction (*F* (3, 87) = 3.90, *p <* 0.05, *η*^*2*^_*p*_ = .119). Post-hoc analyses demonstrated a significant increment on number of breathing cycles from the first interval to the rest of the time windows on both VFTs, being the phonemic task the one displaying the highest values. Analyses of simple main effects indicated that during the first (*p <* .001) and third interval the tasks differed significantly (*p <* .05). As already mentioned, the respiratory rate in phonemic VFT increased from the second interval, however this increment did not reach significant level. It was only on the third (*p <* .01) and fourth (*p <* .05) intervals that the increment achieved significant difference. As for semantic VFT, the rate of respiration significantly raised from the second interval, and it maintained consistent values throughout the remaining intervals. These data are portrayed in Fig 4.

#### Repeated measures correlation

To explore whether total number of correct responses were coupled with changes in airflow measurements at the within-subject level across the different intervals, we conducted repeated measures correlations. Results are presented in Table 1.

**Table 1:** Repeated measures correlations scores between respiratory parameters and VFTs.

Variable	Phonemic fluency correct words	Semantic fluency correct words
**Airflow Duration**		
**Inspiration**	*r*_rm_ (89) = -0.08, *p =* 0.10	*r*_rm_ (89) = -0.18, *p =* 0.10
**Expiration**	*r*_rm_ (89) = 0.17, *p* = 0.46	*r*_rm_ (89) = 0.21, *p =* 0.05*
**Airflow Peak**		
**Inspiration**	*r*_rm_ (89) = -0.44, *p <* 0.001***	*r*_rm_ (89) = -0.51, *p <* 0.001***
**Expiration**	*r*_rm_ (89) = 0.11, *p =* 0.28	*r*_rm_ (89) = 0.09, *p =* 0.39
**Airflow Volume**		
**Inspiration**	*r*_rm_ (89) = -0.16, *p =* 0.14	*r*_rm_ (89) = -0.09, *p =* 0.42
**Expiration**	*r*_rm_ (89) = -0.25, *p =* 0.02*	*r*_rm_ (89) = -0.32, *p <* 0.01**
**Respiratory rate**	*r*_rm_ (89) = -0.26, *p =* 0.01*	*r*_rm_ (89) = -0.49, *p <*0.001***

Note. *r*_rm_ = repeated measures correlation coefficient

* *p <* 0.05

** *p <* 0.01

*** *p <* 0.001

**Fig 5:**
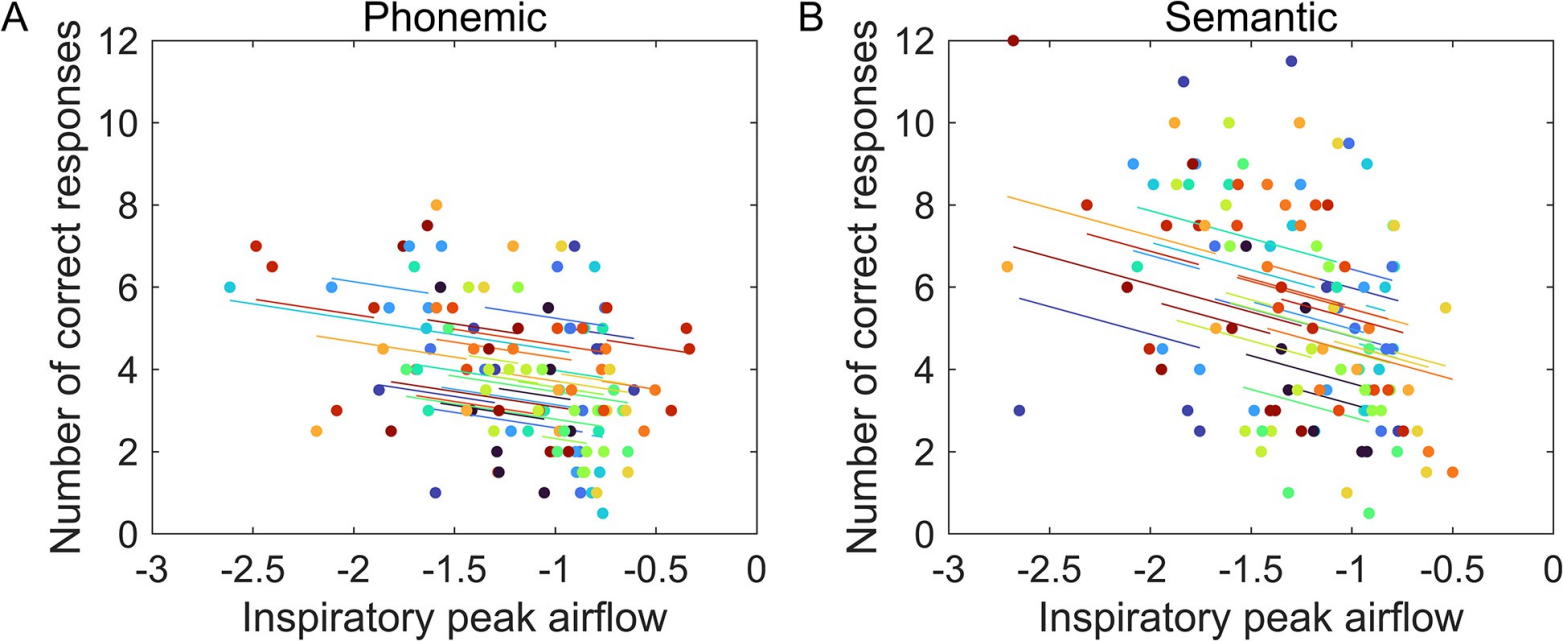
Significant repeated measures correlations between peak inspiratory airflow and correct number of words. A) phonemic and B) semantic VFT. The x-axis shows the values for peak airflow in liters/second while the y-axis shows the mean value of correct number of produced words. Results depict individual data. Each color represents a different participant.

These data showed that number of correct words were significantly associated with changes in peak airflow during inspiration in both phonemic (*r*_rm_ (89) = -0.44, *p <* 0.0001, 95% CI [-0.588, -0.251]), and semantic (*r*_rm_ (89) = -0.51, *p <* 0.0001, 95% CI [-0.651, -0.345]) VFTs. These specific correlations were negative and moderate in magnitude as well as the highest observed (see Fig 5A and 5B).

**Fig 6:**
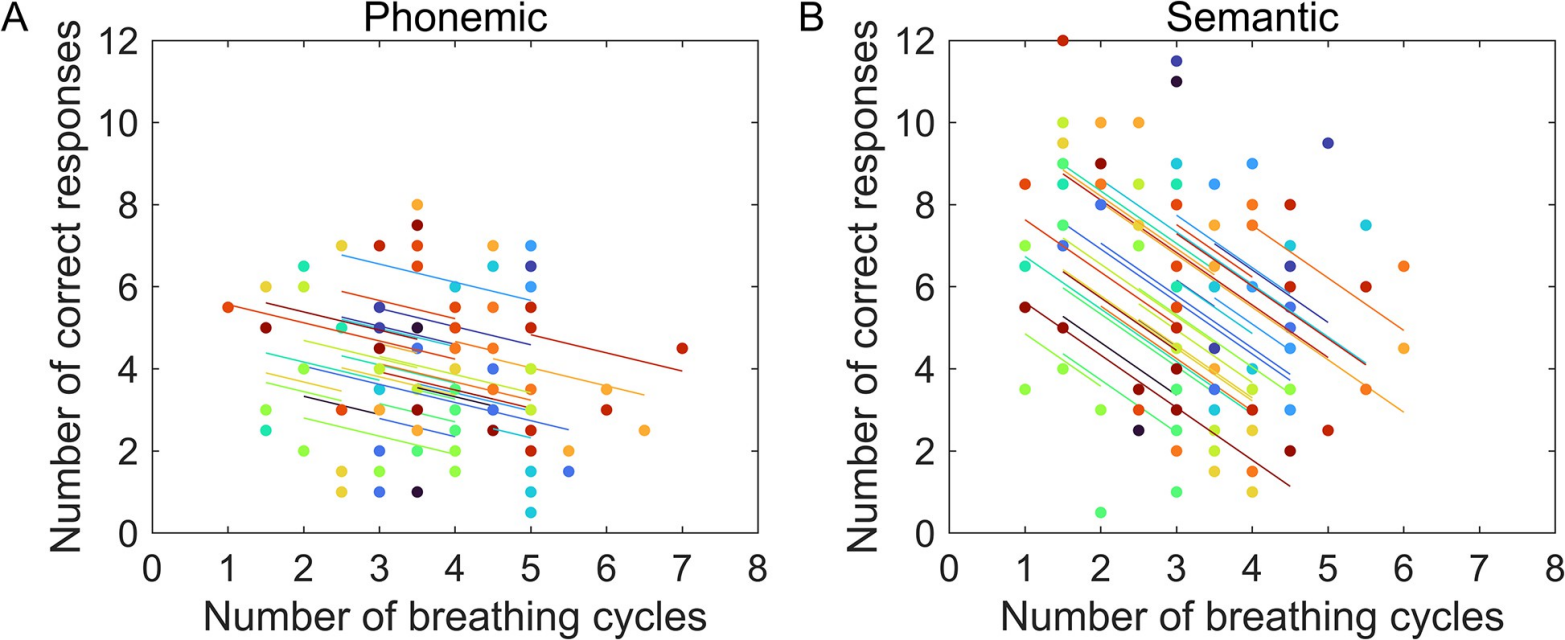
Significant repeated measures correlations between respiratory rate (breathing rate) and correct number of words. A) phonemic and B) semantic phonemic VFT. The x-axis shows the values for mean number of respiratory cycles while the y-axis shows the mean value of correct number of produced words. Results depict individual data. Each color represents a different participant.

However, additional small significant correlations were also found mainly for semantic fluency during expiration. These data showed that increased airflow duration (*r*_rm_ (89) = 0.21, *p* < 0.05, 95% CI [-0.0001, 0.395]) and volumes of air (*r*_rm_ (89) = -0.32, *p <* 0.01, 95% CI [-0.494, -0.123]) were associated with higher number of correct answers during expiration. Also, a small correlation existed during the expiratory phase, between higher airflow volumes and correct answers in phonemic VFT (*r*_rm_ (89) = -0.25, *p <* 0.05, 95% CI [-0.095, -0.311]). A final significant correlation concerned total number of correct responses on both VFTs and respiratory rate. This association was in both cases negative, though for phonemic VFT the link was weaker (*r*_rm_ (89) = -0.26, *p <* 0.01, 95% CI [-0.439, -0.053]), than for the semantic task (*r*_rm_ (89) = -0.49, *p <* 0.001, 95% CI [-0.631, -0.315]) (see Fig 6A and 6B).

## Discussion

The present study was conducted to understand the role of respiration during execution of verbal fluency tasks. The core questions were whether the decay over time in word production during VFT and the discrepancy in output size between VF tasks were related to respiratory function. Results show common respiratory patterns for both VFTs, as well as distinctive breathing characteristics for each VF variant. In order to appraise these findings, we will discuss separately the data related to the decay in performance and the difference in output size between VFTs.

### Role of respiration on the decay over time in VF production

Because we previously reported [13] that specific respiratory traits existed for VF as compared to other verbal tasks during the first 15 seconds of performance, the present study intends to answer whether respiration also varies beyond this interval. Indeed, data showed that breathing patterns changed from the second interval in terms of reductions on peak and airflow volume during inspiration, together with increments in respiratory rate and exhaled airflow volume. From a general view, these data are in line with early research measuring respiratory parameters during execution of non-verbal effortful tasks, such as arithmetic operations or recognition of audiovisual stimuli [41, 42]. Those studies have reported higher respiratory rate during performance of the cognitive tasks and a specific pattern on airflow duration consisting in shorter inhalations and longer expirations. However, by addressing tasks such as VFT that are known to show a decay in performance, we are able to add a new piece of information to this line of study since we can describe respiratory changes as a function of time. Indeed, the pattern on airflow duration and peak airflow as well as respiratory rate during VFTs showed interesting outcomes. For example, airflow duration predominantly remained unchanged across the 1-minute trial, independently of the number of correct words produced. In contrast, respiratory rate increased from the second interval and prevailed throughout the rest of the execution. This suggests that neither airflow duration nor respiratory rate are breathing aspects reflecting the fluctuations in word output. As for peak airflow, significant findings were observed only during inspiration. It was found that after the first 15 seconds, there was a reduction in peak airflow on both VFTs throughout the rest of test performance at the same time that task differences were observed.

A close examination of these data by time interval shows that common readjustments on the mentioned parameters (i.e., airflow volume, inspiratory peak and respiratory rate) takes place after the first 15 seconds of performance. Interestingly, once the readjustments occur, the values of these parameters remain at similar levels of activation until the end of the trial. Statistically, the 2^nd^ and 3^rd^ intervals seem to be more affected, possibly due to the combination of keeping word production at a certain rate but with increased cognitive effort. Conrad & Schönle [37] reported that depending on the degree of language production and difficulty, the respiratory pattern will accordingly be adjusted. Thus, in line with this study we believe that the reason for obtaining significant differences on the 2^nd^ and 3^rd^ intervals is due to a greater effort in word searching, together with superimposed requirements on phonomotoric programs to articulate words.

Thus, the breathing adjustments observed as word production decays along the 1-minute trial, suggest a physiological adaptation to the increment in cognitive effort occurring as a result of exhaustion on the word reservoir. This interpretation aligns with the theoretical framework proposing that degree of effort is related to level of arousal [43]. Moreover, since respiration is linked to the neuromodulation of arousal [44] this finding supports one of our working hypotheses about the use of higher respiratory needs as a consequence of cognitive effort. In terms of a decay in verbal fluency, the above findings suggest that both types of VFT exert common demands on arousal to accomplish performance.

### Role of respiration on the differences in output size between VF tasks

In spite of common respiratory patterns related to a decay in VF performance, we also found specific breathing patterns for each VF test. In fact, we observed that duration and peak of airflow differentiated semantic from phonemic fluencies. During inspiration, we found a small but constant significant difference between tasks across all 4 intervals regarding airflow duration. The data showed that slightly longer inspirations characterized phonemic VFT. As for expiratory duration, task differences were only observed on the last interval, where phonemic VFT displayed a shorter exhalation. These data are intriguing. As referred in the previous section, different accounts in the literature [37, 38] have described that speech production affects in a very specific way the duration of airflow. From a general viewpoint, the characteristics of speech breathing are short inspirations and longer exhalations. This pattern is explained by active and passive forces involving abdominal muscles and the diaphragm [45]. When inhalation occurs, the abdominal muscles and the diaphragm contract, creating a vacuum that promotes air entrance into the lungs. When exhalation occurs, additional voluntary muscles (i.e., intercostal muscles) and the glottis are activated, and air removal is promoted with greater force and control than during inspiration [46]. The amount and pace on which these processes take place are under voluntary control and therefore changes in the use of airflow are connected to the neural planning and execution of speech [47].

In the present study, the usual pattern in airflow duration (i.e., short inspirations and larger exhalations) was replicated. However, differences between tasks were found in which phonemic VF had slightly significantly larger inspirations than semantic fluency across all intervals. Even though the difference in inspiratory duration between VFTs can be regarded as minor, this was a constant occurrence all along the 1-minute trial and independent of number of words generated. In this regard, we remind the reader that word output in phonemic VF is always more restricted than in semantic VF [19]. Therefore, we think that the difference in inspiratory duration might be related to the higher cognitive constraints demanded in the phonemic VFT. This finding means that the inspiratory phase is of importance for word production in VFTs. Herein, we are not the first suggesting that inhalation is related to higher cognitive demands. Perl et al. [48] reported that execution during a visuospatial task was substantially enhanced during inhalation. These authors concluded that inspiration promotes neural activity due to increased attentional demands, which optimizes task performance. In line with this finding, more recent results also have shown that inhalation improves perceptual awareness and decision making [49].

In fact, during the inspiratory phase we observed significant differences both along the trials and between tasks. Notwithstanding, the coherence in the data across breathing parameters during inhalation and task execution is not straightforward. Differences on inspiratory peak airflow between tasks were observed on the 2^nd^ and 3^rd^ intervals. This time, the phonemic VFT showed lower peak levels than the semantic task. If we were inclined to interpret that higher values in the use of airflow during inspiration are related to task difficulty, then peak airflow data should also be greater in phonemic VFT, but it is not the case. Instead, the semantic task displayed significantly higher values for peak airflow, which would signal that either semantic VFT is also a demanding task in terms of cognitive effort or that the more copious production of words in the semantic test requires higher peak of airflow.

Interestingly, inspiratory peak was already remarked in our initial study [13] as having an important link with VFTs, but then we observed increments on peak airflow at *both* phases of the respiratory cycle in both VF tasks. In the present data, adaptations on peak of airflow occur only during inspiration and they are larger in the semantic VFT. In order to interpret these data, the work of Winkworth et al. [47] needs to be mentioned. These authors demonstrated that deeper inspirations precede semantically complex sentences and that the volume of inhaled air together with the peak and the duration of inhalation were parameters related to the planning of an utterance. Based on the above, it is plausible that peak airflow is a parameter that better relates to semantic planning, while the duration aspect during inspiration seems to be of more relevance for phonemic VFT.

Nonetheless, an alternative interpretation by taking into account both results for duration and peak airflow is that a larger peak airflow is needed for breathing deeply and maintaining enough air to produce several words in a short time. This rationale suits data from semantic VF, since a larger number of words is quickly generated while showing higher peak airflow. For phonemic VF, the pattern of adaptations somehow reverts, suggesting that some additional undergoing processes require slighter larger time and slighter lower peak of airflow. All in all, both parameters appear to characterize the conjoint constraints needed for vocalization and cognitive retrieval of words during VF performance. Interestingly and against our expectations, the volume of airflow did not differ between VFT tasks, which indicates that this parameter is not relevant to distinguish VFTs. This is an interesting finding since various past reports including our previous study [13, 36, 50], suggest that airflow volume is significantly related to linguistic factors of a task.

Due to the assorted findings on airflow data, we cannot conclude from them which of the VFTs is the most demanding. However, findings on respiratory rate may help solve the issue. It is the case that respiratory rate was significantly higher for the phonemic task at the first and third interval. The large difference on this parameter (not reported in [13]) between tasks at the beginning of test execution, and then again on the third interval, demonstrates a greater activation for phonemic fluency. In this context, it is evident that the phonemic task triggers an increased state of arousal and as such this data supports the notion that phonemic fluency requires greater respiratory demands due to higher cognitive constraints, which again corroborate Kahneman’s idea [43] suggesting that degree of effort is related to level of arousal.

### Association between VFT accuracy and respiratory parameters

Finally, the repeated measures correlations were important to understand the association between accuracy of performance by breathing phase and test interval at the within-subject level. These data showed only one significant association during inspiration, relating negatively correct number of words to peak of airflow. Because inspiratory peak airflow is represented by negative values, the findings indicate that fewer words generated correlate with shorter peaks. Even though the correlation coefficient was higher in the semantic (*r*_rm_ = -0.51_)_ than in the phonemic test (*r*_rm_ = -0.44), both displayed significant values, suggesting that accuracy of execution in both tasks is related to this parameter. The remainder of the significant correlations pertain to the expiratory phase involving airflow volume and duration. However, in spite of the significant correlations, the coefficients reflect rather poor associations and as such, they are of limited interest for the present study. Conversely, the significant correlation between accuracy of performance and respiratory rate is peculiar and it deserves to be mentioned. This association indicates that for semantic VF, higher respiratory rate is associated with fewer number of correct words. Our interpretation is that increased arousal coupled to low accuracy takes place as a stress response when the lexicon cannot be properly retrieved, which is in line with early research reporting that high levels of arousal are detrimental for performance of difficult tasks [51]. The reason as to why this latter association was more evident on the semantic task, is not straightforward. Still, we propose that an answer can be related to the easiness in producing categories versus producing words after an initial letter. In the former, word retrieval occurs in a regular way through semantic associations [20], and when an interruption happens it might be experienced as more stressful than in the phonemic test, just because the habitual task of retrieving meaningful related words gets hampered. In contrast, word retrieval matching initial letters is rather unusual, and this task poses higher levels of difficulty and stress from the beginning to the end of task execution. In other words, higher levels of arousal are always existent in phonemic VFT, while incapacity to produce words in the semantic VFT at the end of the execution increases the arousal state.

### General discussion

Altogether, the above findings demonstrated a complex interplay of breathing needs that varies along the designated performance time in both VFTs. At the same time, we confirmed specific respiratory patterns on each VF task. Yet, the most compelling finding of this study concerns the way in which respiratory outcomes unfold during execution of VFTs in the inhalation phase. To begin with, we found that adaptations in the inspiratory phase are important for both VFTs. These adjustments included small but constant increments in inhalations during phonemic VFT and higher inspiratory peak airflow values for both tasks, but most pronounced during semantic VFT. These findings agree with the idea that inhalation is coupled to higher order mental functions [23, 48, 49] particularly to syntactic elements of speech [50].

Currently the cerebral networks underlying a link between inspiration and cognition have been proposed [52]. Firstly, the brain center controlling inspiratory function is a group of neurons known as pre-Bötzinger complex [53]. This area in the brainstem has direct projections to the locus coeruleus (LC), which has a key role in arousal and acts as a synchronizer of inspiratory rhythms and attention [54]. Thus, the connections linking the pre-Bötzinger complex, and the LC are the neural substrates modulating the interrelation between breathing and cortical excitability [52]. However, because inhalation has been repeatedly linked to cognition, it is suggested that the inspiratory phase particularly enhances attention and optimizes cortical function [48, 55]. In line with the above, our findings also point to an interplay of breathing adaptations during the inhalation phase to properly accomplish VFTs. Therefore, we strongly suggest that the role of inspiration in VFTs must be followed-up by analyzing the variability of inspirations during word production and retrieval, including breath holdings, sights and pauses. In fact, earlier studies have underlined the importance of looking into respiratory discontinuities [37, 56, 57], as these may cast light on ideational processes related to how respiration interplays with word retrieval. Remarkably, the expiratory phase was of no further interest for performance of VF, as it neither showed significant adaptations for word generation across intervals nor it differentiated VF outputs between tasks. Additional investigations need to corroborate this finding.

Besides the important role of inspiration for VF performance, we found a clear general increment in arousal, in terms of respiratory rate, which agrees with early accounts [18]. However, even if this increment was turned on in both VFTs across time, this activation does not necessarily optimize the accuracy of word production. At this respect, we point out the high level of arousal observed in phonemic VFT, which we interpret as a consequence of its higher difficulty, which in turn might also trigger increased levels of stress [18] all along the execution. Another important finding of the present study is the selected impact of individual airflow parameters on VF performance. In fact, only single parameters were coupled with each of the VFTs in the inspiratory phase, namely airflow duration was relevant for phonemic VF, while high peak airflow was particularly higher during semantic VF.

Finally, the issue of directionality in the respiratory-cognition association was not entirely solved since our data demonstrated mixed findings. For instance, our first hypothetical scenario stated that respiratory needs might increase proportionally as a result of cognitive effort while performing VFTs. However, this was true only for respiratory rate, but not for the airflow parameters. In turn, some of the airflow data aligned with the other two proposed alternatives, that is, there were decreases in peak and volume as well as no changes on airflow duration. All things considered, these data show that there is no clean direction in the way respiratory function and verbal fluency performance influence each other. Instead, the relationship seems to be *bidirectional*, which matches theoretical proposals on the dynamical interactions between psychophysical status, cognitive functioning, and breathing [50, 58].

### Limitations and future venues

The present study is not without limitations. As in many VF studies, we conducted the experiment in one language, Norwegian as the mother tongue of our participants, with a limited selection of categories and letters to tests the VFTs. Therefore, exploring additional categories and initial letters for each VF test would help to address how level of stimuli difficulty affects respiratory outcomes. In parallel, it is recommended to reproduce the present investigation in other languages than Norwegian to clarify whether comparable results on breathing requirements can be achieved. As well, the approach used in this study could not reveal which mechanisms govern the directional relationship between breathing and VF, as both functions are interchangeably driven. In order to achieve a better understanding of these dynamics, it would be necessary to carry out approaches in which the control of breathing during VF execution is manipulated.

Another limitation is our restrictive focus on the accuracy outcome (i.e., correct produced words) of verbal fluency. Additional estimations of breathing requirements vis-à-vis other VF parameters such as errors, repetitions, non-related words, and undefined voicing (e.g., filling words, or noises) will complement the present findings. Further inspection of inhalation pauses of all sorts including sighs, will help disentangle the mechanisms behind the interplay between word production in VF and respiratory function. Also, the fact that we did not acquire resting tidal breathing data can be regarded as a limitation. Nonetheless, since resting breathing is too variable between subjects and highly affected by several variables such as age, health, sensory stimulation, motivation, stress, and emotions [44], this condition was regarded as inconvenient for baseline of the study.

Finally, we suggest that future studies addressing the link between breathing and VF performance explore how the potential effects of sex and age impact this association. It is acknowledged that differences between men and women exist in respiratory physiology not only related to anatomy (e.g., women having smaller lungs), but also to sex-hormones and fluctuations in hormonal levels [59]. In addition, there is strong evidence for sexual dimorphism in the prevalence of respiratory ailments at different stages of the lifespan [60]. Conversely, sex differences in VF tasks are still a matter of debate [61]. Some studies report that women outperform men in phonemic VFT (e.g. [19]), while others have failed to observe any sex differences (e.g., [62]). All things considered, addressing the functional significance of sex differences in breathing at specific periods of time (e.g., puberty, menstrual phases, menopause) may eventually help elucidate the mixed results about sex differences in VF ability.

## Conclusions

For the first time, the present study has identified specific breathing signatures of a verbal ability that represents an important aspect of intelligence, and which shows both commonalities and particularities for their test variants. In agreement with previous accounts [44], we were able to assert that the role of breathing during verbal fluency performance cannot be regarded as pure physiological noise, but rather it is an important orchestrator for higher-order brain functioning. In particular, the present study highlights the inspiratory phase of the breathing cycle as a decisive stage moderator of cognitive performance during verbal fluency in healthy young adults. Future investigations should aim to settle the universality of these findings by acquiring verbal fluency profiles of other populations including healthy individuals at different periods in the lifespan.

## Supporting information

S1 Fig Illustration of an acoustic signal (top panel) and corresponding respiratory output (bottom panel) on a phonemic VF task. Note that arrows show the sense of airflow, and examples of duration data in inspiratory and expiratory data are marked. Airflow peaks are shown by dots.
(DOCX)

S1 File Dataset of all variables used in analyses. (XLSX)
