# Supplementary material for: Breathing signatures of semantic and phonemic verbal fluency and their impact on test performance in a sample of young Norwegian adults
Source: PLoS One. 2024 Dec 5;19(12):e0314908. doi: 10.1371/journal.pone.0314908 (PMC11620639; doi:10.1371/journal.pone.0314908)
Supplement: S1 Fig — Illustration of an acoustic signal (top panel) and corresponding respiratory output (bottom panel) on a phonemic VF task. Note that arrows show the sense of airflow, and examples of duration data in inspiratory and expiratory data are marked. Airflow peaks are shown by dots. (DOCX) [file pone.0314908.s001.docx]

**S1 appendix Figure 1**

**
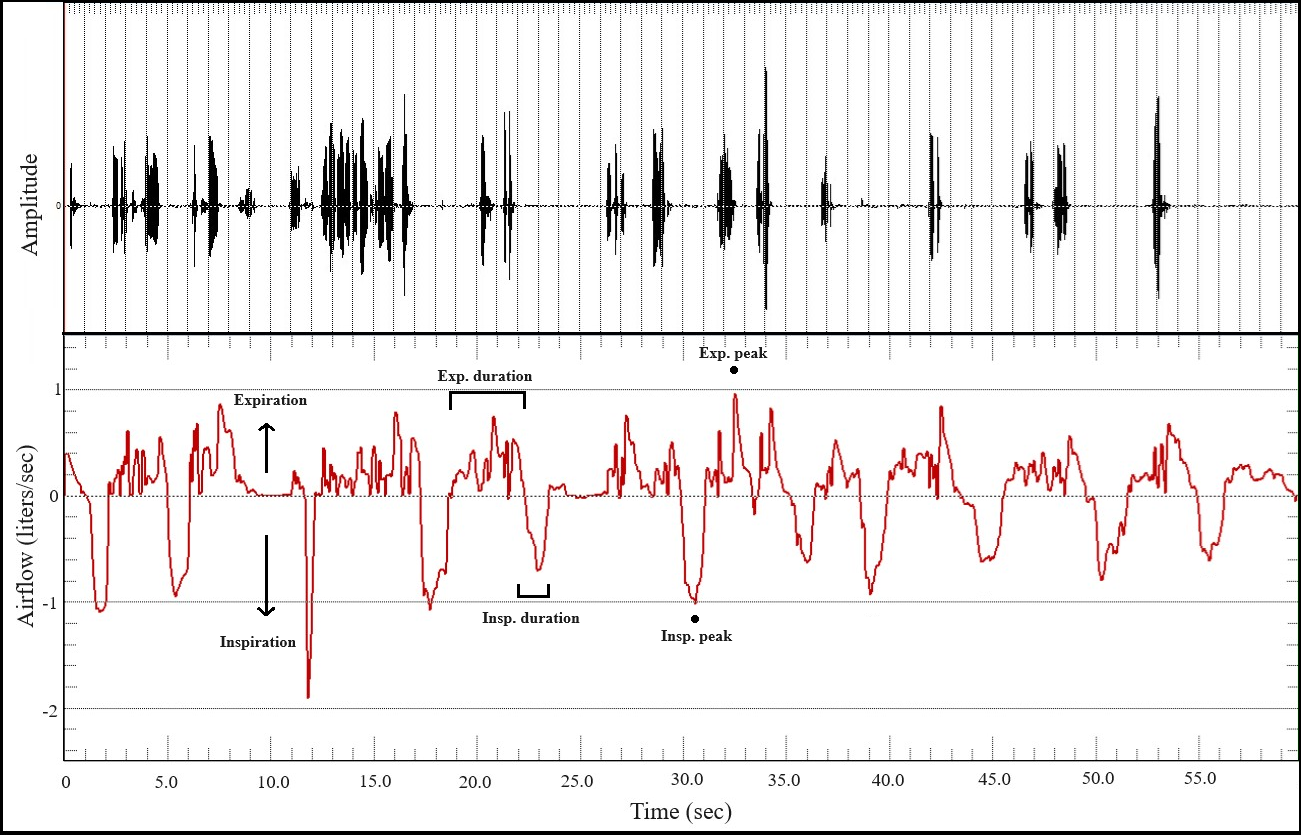
**

Illustration of an acoustic signal (top panel) and corresponding respiratory output (bottom panel) on a phonemic VF task. Note that arrows show the sense of airflow, and examples of duration data in inspiratory and expiratory data are marked. Airflow peaks are shown by dots.
